# Predicting Perceived Exhaustion in Rehabilitation Exercises Using Facial Action Units

**DOI:** 10.3390/s22176524

**Published:** 2022-08-30

**Authors:** Christopher Kreis, Andres Aguirre, Carlos A. Cifuentes, Marcela Munera, Mario F. Jiménez, Sebastian Schneider

**Affiliations:** 1 Faculty of Technology, Bielefeld University, 33615 Bielefeld, Germany; 2 Department of Biomedical Engineering, Colombian School of Engineering Julio Garavito, Bogotá 111166, Colombia; 3 Bristol Robotics Laboratory, University of the West of England, Bristol BS16 1QY, UK; 4 School of Engineering, Science and Technology, Universidad del Rosario, Bogotá 111711, Colombia; 5 Applied Informatics Faculty of Technology, Bielefeld University, 33615 Bielefeld, Germany

**Keywords:** physical activity recognition, affective computing, facial action coding systems, rehabilitation

## Abstract

Physical exercise has become an essential tool for treating various non-communicable diseases (also known as chronic diseases). Due to this, physical exercise allows to counter different symptoms and reduce some risk of death factors without medication. A solution to support people in doing exercises is to use artificial systems that monitor their exercise progress. While one crucial aspect is to monitor the correct physical motions for rehabilitative exercise, another essential element is to give encouraging feedback during workouts. A coaching system can track a user’s exhaustion and give motivating feedback accordingly to boost exercise adherence. For this purpose, this research investigates whether it is possible to predict the subjective exhaustion level based on non-invasive and non-wearable technology. A novel data set was recorded with the facial record as the primary predictor and individual exhaustion levels as the predicted variable. 60 participants (30 male, 30 female) took part in the data recording. 17 facial action units (AU) were extracted as predictor variables for the perceived subjective exhaustion measured using the BORG scale. Using the predictor and the target variables, several regression and classification methods were evaluated aiming to predict exhaustion. The results showed that the decision tree and support vector methods provide reasonable prediction results. The limitation of the results, depending on participants being in the training data set and subjective variables (e.g., participants smiling during the exercises) were further discussed.

## 1. Introduction

According to the World Health Organization, non-communicable diseases are responsible for 71% of the global deaths (41 million approximately) each year, cardiovascular diseases, cancers, respiratory diseases, and diabetes being the most common ones [1]. Physical exercise has become an essential tool for treating various non-communicable diseases (also known as chronic diseases). These exercises allow to counter different symptoms and reduce some risk of death factors without medication, such as hypertension, low cardio-respiratory capability, pathological fatigue, and overweight [2]. Therefore, several rehabilitation programs have incorporated different types of exercise (e.g., walking, riding, vertical jumps, and lifting external loads) into their therapies [3,4,5].

One of the most implemented exercises is the sit-to-stand (STS), due to it being easy to apply in any environment and reflects one of the most common daily life activities [6]. The STS test consists of sitting down and standing up from a chair as fast as possible, during a certain period (normally, between 30 to 120 s) [7], and has been used to treat patients and assess their physical condition in several rehabilitation environments, such as cardiac [8], oncology [9], pulmonary [10] and neuromuscular [11].

Despite the benefits that the STS exercise (and in general the physical exercise) provides, several considerations must be taken into account at implementing it in rehabilitation, due to taking patients to prolonged periods, extreme exercise conditions, and/or high fatigue levels, might lead them to suffer physical or physiological complications [12]. Thus, it is indispensable to monitor the patient’s condition during the physical therapies [13]. To accomplish this goal, several methods have been explored, which can be divided into 3 groups [13].

The first one consists of monitoring physiological parameters related to the user’s physical condition (e.g., heart rate, breathing frequency, temperature, blood lactate level, oxygen saturation, blood pressure, and others) [14,15]. However, some of these parameters require complex instrumentation and can not be measured easily during the execution of the activity, specifically, blood pressure, oxygen saturation, blood lactate level and oxygen uptake [16,17]. Considering the practical scenarios of the rehabilitation programs, the health professionals prefer not to use these parameters in their therapies [16].

On the other hand, some physiological parameters are easy to monitor and provide a good indicator of the patient’s fatigue condition. These parameters usually have a linear relation with the amount of energy expended, like the heart rate or the breathing frequency [18]. Nonetheless, studies have shown that for short-duration and intense exercises (like the STS), these parameters do not present the same lineal behavior and do not change as fast as the patient’s condition. Therefore, it is not recommended to use them as the only indicator for monitoring [18].

The second method is a subjective technique where the patients are asked about their perceived exertion, according to established ordinal numeric scales [19]. One example of this method is the 10 points Borg’s scale (Borg CR10), which is a scale composed of 11 levels (from 0 to 10), where 0 represents no-exertion, and 10 is the maximum effort [20]. Due to its facility to be implemented and understood, the Borg CR10 is one of the most applied techniques in physical rehabilitation [21]. Nevertheless, studies have criticized these subjective methods, because they do not always represent the real intensity compared to the physiological parameters [22].

Finally, the last method is a novel idea based on processing image techniques. In general, the main idea consists of detecting relevant features from patients’ images (e.g., facial features, skin color, and movement analysis) during the exercise, that supply any information about his physical condition [23]. Although this method requires some complex computational process, studies have shown that it is possible to monitor subjects’ conditions with accessible systems during different activities. For example: in [24] the authors developed an image-based system to detect fatigue while the user is driving, just with an RGM infrared camera; in [25], a fatigue estimator model based on thermal images and face analysis is presented, and in [26] the authors used the depth and RGB images from a Kinect sensor, to assess the users’ condition in home-based exercises.

However, to the author’s knowledge, this image-based method has not been applied to the STS exercise, despite this activity being performed in a controlled area, which facilitates the implementation of this technique. Therefore, taking into account: (i) the importance of preventing patients from extreme exercise conditions, (ii) that there is a need for more monitoring techniques, and (iii) the use of the STS exercise in the rehabilitation programs; the aim of work is to develop an image-based system for predicting the user’s perceived exertion during the STS exercise.

For this purpose, we have collected a new data set of participants doing STS exercises and recorded them during the exercises. Our goal of this work is to use this data set to test different machine learning models, both regression and classification models, that predict a participant’s subjective exhaustion based on extracted facial action units (AUs). This methodology has comparative advantages to previously used methods. First, the user does not have to wear any additional sensors. Second, since only a camera system is needed, it can be easily deployed and even be integrated into artificial exercising companions that monitor the performance of a trainee (e.g., social robots).

The document is organized as follows. Section 2 introduces related work on the facial action coding system and previous studies where action units are used to identify muscle fatigue. Section 3 highlights the methodology for our data set collection. Our recognition pipeline is explained in Section 4. The evaluation of our models is highlighted in Section 5 and followed by a discussion in Section 6. The paper ends with a conclusion on our achieved results.

## 2. Related Work

### 2.1. Facial Action Coding System

The Facial Action Coding System (FACS) was developed by Paul Ekman and Wallace V. Friesen with the aim to formalize a uniform and understandable system to record facial expressions. This system should be able to represent all human facial movements and therefore be applicable across disciplines. To be universally applicable, the FACS only records facial expressions and does not make any statement or interpretation about the face, such as the emotions conveyed by the facial expression [27].

In the development of the FACS, Ekman and Friesen examined how the activation of various facial muscles affects facial expression. Based on their observations, they developed various Action Units (AU) that describe the different movements in the face. These action units are minimal in the sense that the facial movement cannot be further divided. The AUs are based on the anatomical principles of the face, but it should be noted that not every AU represents a single facial muscle. Some AUs represent a group of muscles because different muscles cause the same change in the face, or the changes are so similar that they cannot be reliably distinguished. In other cases, a muscle could cause different, independent movements on the face [27].

The FACS comprises a total of 44 different AUs. Thirty of these AUs relate to specific muscles or muscle groups. The remaining fourteen AUs describe diverse behaviors that cannot be attributed to specific muscle groups, such as lip biting. While some AUs exist or do not exist, other AUs can occur at different intensities. These AUs are divided into five different intensity groups (cf. [28]).

### 2.2. Using Action Units to Identify Muscle Fatigue

Uchida et al. [23] looked at how facial expressions of a person doing strength training exercises change with increasing fatigue. The study had 11 male participants who were 23 years of age on average (the standard deviation was ±6 years) and had been doing strength training for at least 12 months before the study.

The study consisted of two sessions for the participants. At the first appointment, general data such as weight and height were recorded. In addition, the one-repetition maximum (1RM), the maximum weight with which an exercise can be carried out once, was determined for bicep curls with a barbell. On the second date, the actual exercises took place, in which the data were recorded. After the participants had warmed up, they performed the biceps curls with the maximum number of repetitions possible with 50% and 85% of the 1RM. The participants took a ten-minute break between the exercise with 50% of the 1RM and the exercise with 85% of the 1RM. During the exercises, the participants were filmed frontally with a video camera, with the focus on the face.

The video recordings were manually evaluated by five researchers who had experience with strength training and the facial action coding system. AU12, which is raising the corners of the mouth, was determined for the first and last repetition of the exercise. Each subject was rated five times.

Uchida et al. [23] found that the values of the AU12 during the first repetition were significantly different from the values of the last repetition. This difference was significant both in the exercises with 50% of the 1RM and in those with 85% of the 1RM. The value of AU12 increased by approximately 76% in the exercise with 50% of the 1RM and approximately 66% in the exercise with 85% of the 1RM.

The authors conclude from the observations that there may be a direct correlation between a person’s facial expression and exhaustion. Furthermore, the authors suspect that AU12 could be an indicator of exhaustion [23].

Another study analyzed facial action units during ergonomic cycle training [29]. The authors recorded 132 individuals while they performed an incremental test on a cycle ergometer. They recorded perceived exhaustion as well as affective valence every two minutes during the training. For their evaluation, the authors used the commercially available Affectiva SDK to extract AUs and used multi-level regression analysis to investigate how perceived exhaustion and valence covaried with the AUs. Their main finding showed that mouth open and jaw drop predicted the subjective rating for perceived exhaustion. This finding suggests that these AUs are activated due to the physiological need for optimized gas exchange in the working organism.

The above-mentioned projects are the only known related works that connect the facial action coding system with the exhaustion caused by sporting activities. In contrast to the work by Uchida et al. [23], the AUs in this work should be calculated automatically and not determined manually. In addition, the aim of this work is not to investigate the relationship between AUs and exhaustion, but to develop a predictive model. This model should not only consider the AU12 for forecasting but also include a large number of AUs in the decision.

## 3. Methodology

### 3.1. Subjects

For the experiments, 60 participants distributed in 30 females and 30 males were recruited to perform a 2 min sit-to-stand test. In addition, the inclusion criteria were as follows: healthy adult subjects between 18 to 30 years old, weighing between 50 to 75 kg, and without any fatiguing condition according to the “multi-dimensional fatigue inventory”, which is a 20-item questionnaire to estimate 5 different types of fatigue states (general fatigue, physical fatigue, mental fatigue, reduced motivation, and reduced activity) [30]. Subjects with physical impairments that impede the transition from sitting down to standing up, cognitive impairments that do not allow to follow instructions, conditions that pose a risk for a participant in a fatigued state, and/or use prostheses or orthosis in their limbs, were excluded from this study. We used these inclusion criteria because we needed to guarantee no harm or feeling of uncomfortableness to people. Thus, we had to include only healthy subjects who could perform the exercises for our experiments. Before starting the experiment, the volunteers signed the informed consent to accept participation in this study. Table 1 shows the mean and standard deviation (M ± SD) of volunteers’ demographic data, for the female (f) and male (m) groups.

### 3.2. Materials

To obtain the STS images, the heart rate, and the fatigue level of each volunteer, a multitasking application was developed. Such applications incorporate and synchronize the following tools in a single computer process:

- For STS images was used a Kinect V2 (Windows, USA). This Kinect provides images with a depth of 512 × 424 pixels of resolution and RBG of 1920 × 1080 pixels of resolution. Besides, it was used a tripod with a height of 1 m from the floor and 4 m of distance from the subject to locate the Kinect.
- The heart rate was measured with a Zephyr HxM BT (Medtronic, Ireland) sensor. The sensor used Bluetooth communication to transmit the data with a sample rate of 1Hz. This sensor was placed on the volunteer’s chest with an elastic band.
- Borg CR10 scale was used to evaluate the test. This scale was asked to the volunteer every 30 s during the STS test. This way, a total of 4 Borg CR10 values were obtained if the volunteer was able to complete the 2 min exercise.

**Figure 1:**
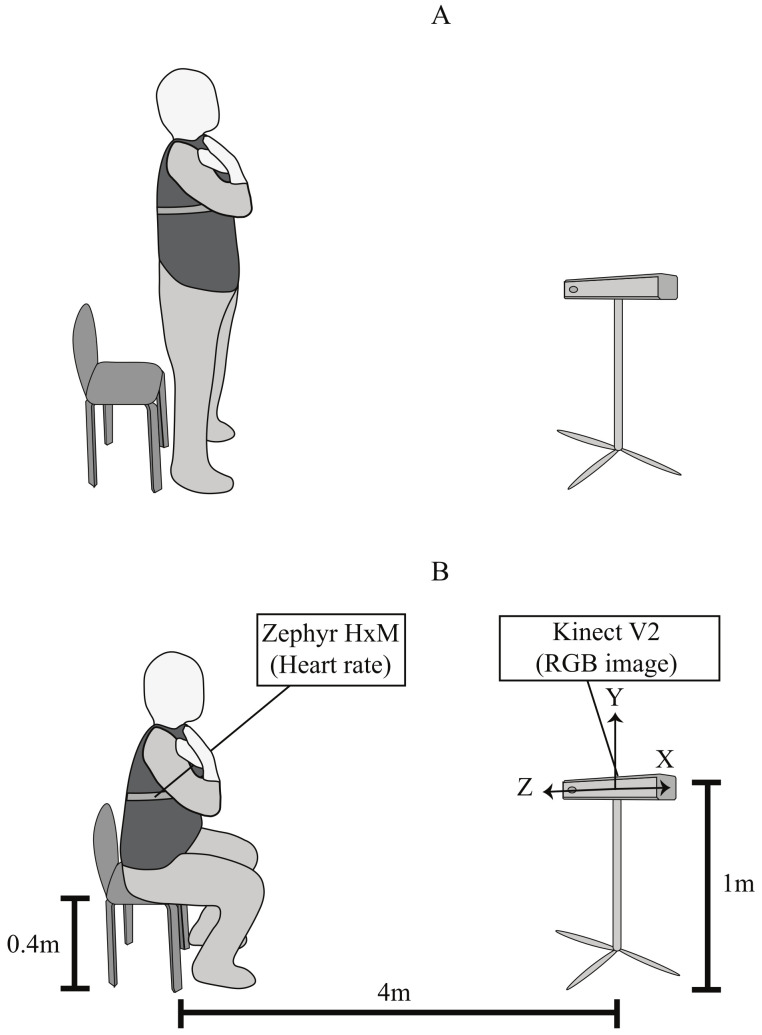
Set-up of the study and sit-to-stand representation, (**A**) standing position and (**B**) sitting position.

The STS exercise representation and the study set-up can be seen in Figure 1, where Figure 1A shows the standing position; and Figure 1B illustrates the sitting position and the sensor location.

### 3.3. Experimental Procedure

**Figure 2:**
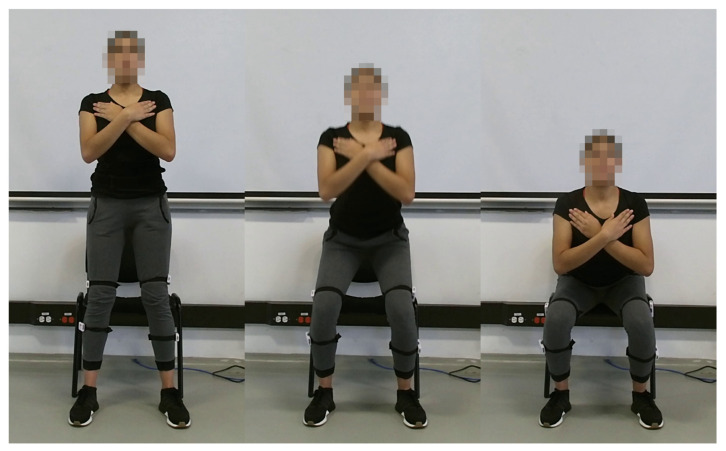
Recording sample.

As the first step, it was estimated the maximum heart rate (MHR) of participants through the “Tanaka equation”, which is shown in Equation (1). In this equation, the MHR is calculated according to the subject’s age [31]. It was used a test that consisted in sitting down and standing up from a chair as fast as possible for 120 s (2 min). To start the test, the participants stay in a standing up position and begin after listened the command “go”. The participants were asked to maintain their hands on their shoulders, and look straight forward during the experiment (see Figure 2). The test was considered concluded if Borg Scale registered a value of 10, or if the sensor reported a heart rate value higher than 90% of the participant’s MHR. After the test, the participants were required to do a 5 min cold-down.

(1) MHR=206.9−(0.67×AGE)

## 4. Exhaustion Recognition Pipeline

The main goal of our work is to investigate whether we can predict subjective exhaustion during physical exercises based on facial expression. This section introduces the general classification pipeline, the required pre-processing, the model training, and evaluation.

**Figure 3:**
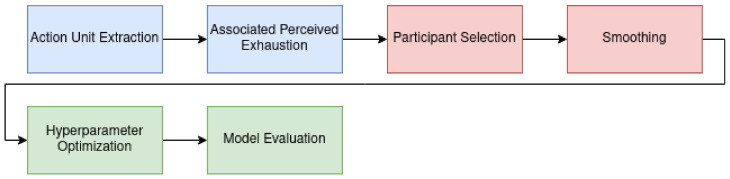
Data processing pipeline consists of an Action Unit (AU) Extraction from videos using OpenFace. The AUs are stored together with the participant’s subjective perceived exhaustion on the Borg10 scale. Following, participants are manually assigned labels regarding whether they are smiling and whether they subjectively look exhausted. The time series is then smoothed using various filters. Finally, hyperparameters are tuned using a validation set, and the model is evaluated.

Our learning pipeline is divided into three parts. In the first step of the learning pipeline (blue boxes in Figure 3) computations are only carried out once. These computations include extracting the AUs from the video files. Accordingly, the Borg values were then estimated using a linear model based on the AU timestamps. Since these are the features for all models in our work, we calculated data once and stored them permanently. The second step of the pipeline (red boxes in Figure 3) consists of parameter-wise calculations. These computations were recalculated depending on the specific setting and took place before the actual model training. We selected only subjects that met specific qualitative criteria. After we selected the data for the training, the calculated AUs were smoothed with one of four filters. The actual model training takes place in the last part of the pipeline (green boxes in Figure 3). Hyperparameters were optimized by grid search with cross-validation. After dividing the data into a test and a training data set, scaling was applied to the training data in the individual steps of the cross-validation, followed by the principal component analysis (PCA). Finally, cross-validation was carried out on the entire data set using the determined hyperparameters to evaluate the models.

### 4.1. Data Set Development

As described in Section 4, the features were extracted from the experiment data. This subsection explains how the data set was created and what data it contains.

#### 4.1.1. Video Data Processing

Before we processed the video data, we cleaned it from the noise that could disturb our analysis (e.g., people in the background). This ensures that only the face of the test person can be seen in the video so that it is not necessary to distinguish which face the calculated action units belong to.

#### 4.1.2. Extraction of Action Units

We calculated the AU data using OpenFace. OpenFace provides functions that extract action units from an image or video [32], and can calculate the intensity of 17 different AUs, which are listed in Table 2. A support vector regression with a linear kernel is used for this. This was trained on several different data sets, including DISFA, BP4D-Spontaneous, and SEMAINE [32].

In addition to the static extraction of action units from images, OpenFace also implements dynamic extraction from video data. The neutral facial expression is learned by a person, which increases the accuracy of the calculated AUs [32]. OpenFace requires about 300 images of a neutral face for learning. Since the test subjects usually have a neutral face at the beginning of the exercise, the beginning of the video recordings is suitable for this. It should be noted that the neutral face is continuously optimized, while at the same time the AUs are calculated for the current image. Two different approaches were therefore considered in this work. In the first approach, the first 300 pictures were used to train the neutral face. The video file was read in again for the calculation of the AUs, so that a neutral face was also present in the calculations based on the first 300 images. In the second approach, the video file was only read once, so that there is still no neutral face when calculating the AUs of the first 300 images. The effects of this on the trained model are extremely small. Therefore, we used the version without renewed training. This also has the advantage that it can be used in a real-time application.

After calculating and exporting the AUs for all videos, we merged the data with participants’ subjective ratings. Since the created model should predict subjective exhaustion, those data points were extracted from the subjective ratings for the Borg CR10 value. Four Borg ratings for each participant were obtained. Although there were also some records that only contained two or three data points with the Borg value. As the Borg values in our data set approximate a straight line on the time scale, we decided to approximate the Borg values using a trend line. The values for the individual AU data points were calculated using that trend line. For normalization to the interval [0, 1], the calculated Borg values were divided by ten. The other entries in the table data, such as different joint positions, are not relevant for this work and were therefore not considered further.

Since we want to train a model for classifying exhaustion, we devise the classes as *exhausted* and *not exhausted*. For this, data points whose standardized Borg value was greater than 0.5 were assigned to the class *exhausted*, while the remaining data points were assigned to the class *not exhausted*. This limit was chosen because a five on the Borg CR10 scale corresponds to severe fatigue [22].

### 4.2. Data Set Preprocessing

Before training the model, the data set is pre-processed. Such pre-processing includes the selection of data that is used to train the model and the subsequent smoothing of the AUs.

#### 4.2.1. Selection of Participants

When the video files were processed, it was noticed that the behavior of the different participants are distinct. While some participants had strained facial expressions at the end of the exercise, others had neutral facial expressions. Furthermore, some participants started to grin during the activities, which is probably due to the unusual situation of the test scenario. Since a model could have difficulties with the participants who react very differently to the effort, it was tested with a selection of data to improve the predictions. For this purpose, different values were assigned to the images around the smile. The parameter *smiling* indicates how much the subject grins when he/she performs the exercise. This parameter takes on values from zero to two, and its meaning is listed in Table 3.

The rating parameter evaluates how meaningful the subject’s facial expression is during the exercise. A high value means that the exhaustion is easily recognizable. Low values, in turn, reflect unrecognizable or poorly recognizable fatigue. The list of the different values can be found in Table 4.

The selection of the data set was made based on the two parameters aforementioned, so that in the following steps, only recordings are considered whose *smiling* and *rating* parameters lie within a defined interval. For example, only people who do not grin when performing the exercise and who look slightly or very exhausted at the end of the activity can be considered.

#### 4.2.2. Action Unit Smoothing

**Figure 4:**
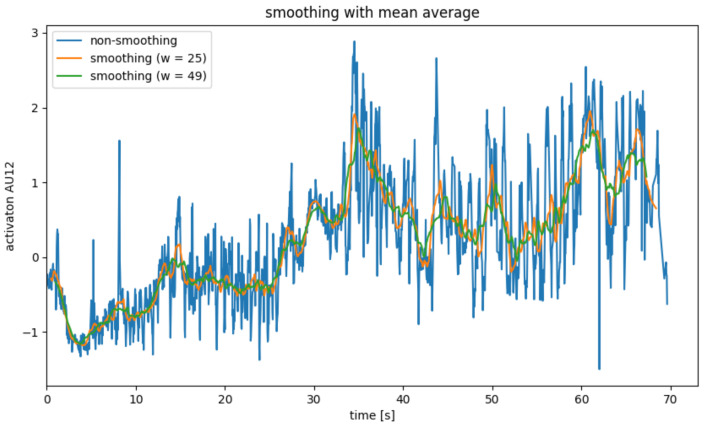
AU smoothing with average mean filter.

**Figure 5:**
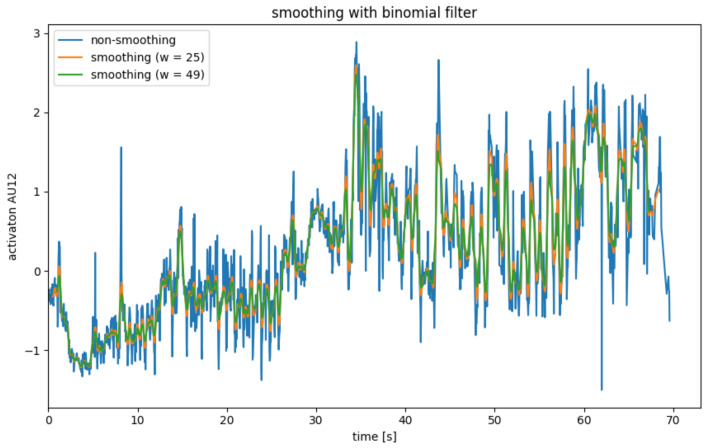
AU smoothing with average mean with binomial filter.

**Figure 6:**
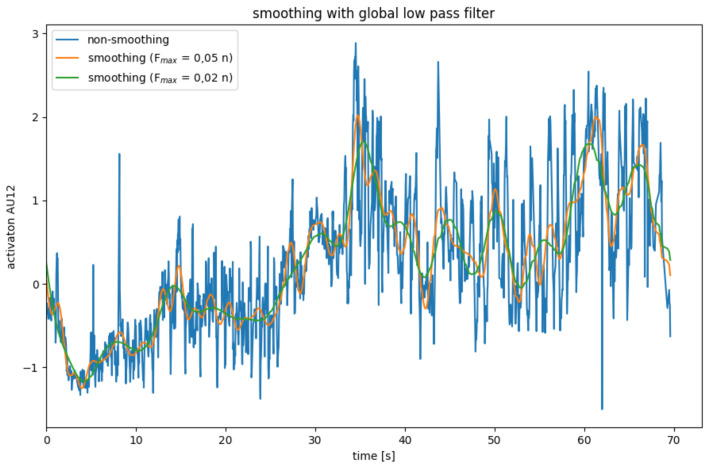
AU smoothing with average mean with a global low pass filter.

**Figure 7:**
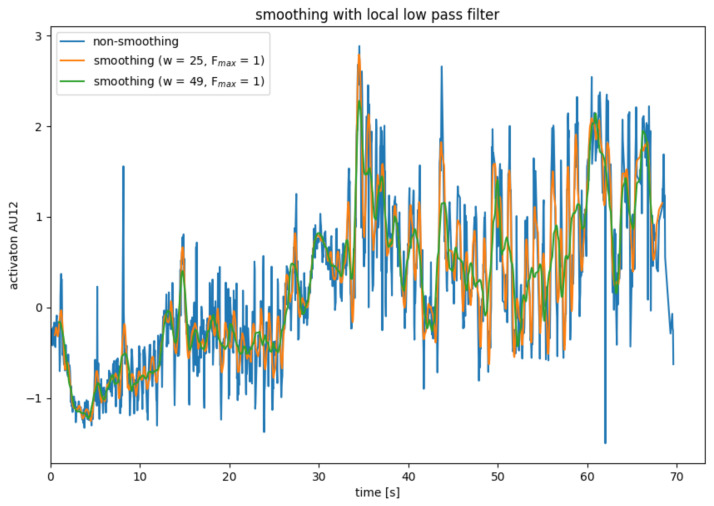
AU smoothing with average mean with a local low pass filter.

Since the AU data calculated in the previous step is very noisy, it is necessary to smooth it in a pre-processing step. For this, four different smoothing operators were considered, which will be explained in Figure 4, Figure 5, Figure 6 and Figure 7.

##### Moving Average

The mean filter, also called the moving mean, smoothes a value $x_{i}$, by forming the mean over $x_{i}$ and its neighboring values. This corresponds to folding with the folding core $\frac{1}{w}\left(\begin{array}{cccc} 1 & 1 & \cdots & 1 \end{array}\right)$, where *w* is the width of the folding core. To ensure that as many neighbors are viewed on both sides by $x_{i}$, convolution kernel with an odd width is generally used [33].

Since the same number of neighbors on both sides is required to smooth $x_{i}$, the moving average cannot be used for the marginal areas of the value series. As a result, data points are lost due to the smoothing of w−1.

In addition, the mean value filter only looks at the local environment of a data point, so it can also be used in applications in which data arrive in real-time. However, this results in a delay corresponding to the time it takes for $\frac{w-1}{2}$ to receive more data points because $\frac{w-1}{2}$ neighboring points on both sides of $x_{i}$ are needed to smooth $x_{i}$.

Figure 4 shows the smoothing of AU12 for a single subject with an average filter. The width *w* was chosen as 25 and 49, respectively, since the video recordings were recorded at a frame rate of 25 fps. As a result, the delay caused by the filter is approximately 0.5 s or around 1 s. It becomes clear that the mean value filter is well suited to smoothing out the steady noise of the calculated AUs (see Figure 4, blue graph). Here, the strength of the smoothing and the delay caused by the smoothing must be weighed up.

##### Binomial Filter

The binomial filter, which is often referred to as the Gaussian filter, uses a convolution just like the mean filter to smooth the point $x_{i}$. However, the binomial filter uses a different convolution kernel, which weights the neighboring points from $x_{i}$ based on their distance to $x_{i}$. The binomial coefficient can be used to generate a convolution kernel of order *k*, which has the width w=k+1 [33].

Bk=12kk0k1⋯kk

Like the mean filter, the binomial filter has the problem that edge data points are lost and that there is a delay.

The application of a binomial filter is illustrated in Figure 5. The lower weighting of the more distant neighboring points is evident from the fact that the peaks are weakened significantly less than with the mean filter. In this application, the mean filter is, therefore, preferable to the binomial filter.

##### Global Low Pass Filter

Another approach to smoothing data is to use a low pass filter. Here, the data are transformed into the frequency domain using a Fourier transformation. A data series with *n* data points can be represented by overlaying *n* frequencies. The low pass filter leaves low frequencies below a cut-off frequency $F_{max}$ unchanged, while high frequencies are set to zero. Since high frequencies mainly represent noise, while the actual signal predominates in the lower frequencies, the data is smoothed by cutting off high frequencies. After the data series have been smoothed in the frequency domain, it is transformed back into the spatial domain through an inverse Fourier transformation [33].

This method has the advantage that no data points are lost because there are no border areas. However, it can only be applied to complete data series and is therefore not suitable for real-time systems.

The effects of the global low pass filter are illustrated in Figure 6. The limit frequency $F_{max}$ is determined depending on the number *n* of data points. The resulting smoothing is better than that of one of the binomial and mean filters.

##### Local Low Pass Filter

The low pass filter described in the previous subsection has the disadvantage that it works globally on the entire data series. Similar to the mean value filter or the binomial filter, the low-pass filter can be adapted so that it only works on a section of the data. To smooth data point $x_{i}$, a normal low-pass filter is applied to data points $x_{i-k}$ to $x_{i+k}$. Only the data point $x_{i}^{\prime }$ is used from the smoothed partial data series. For the remaining data points of the partial data series, other partial data series are used for smoothing.

Due to the Fourier transformations, the local low-pass filter is significantly more computationally complex than the mean filter and the binomial filter. Compared to the global low-pass filter, the local low-pass filter has the disadvantage that data points are lost during smoothing and that there is a delay.

Figure 7 shows the use of a local low-pass filter, whereby only the lowest vibration is obtained when smoothing the partial data series. The results of the local low-pass filter are slightly better than those of the mean filter but significantly worse than those of the mean filter.

### 4.3. Model Training

This subsection explains how the different tested models were trained. For this purpose, it discusses the models firstly, and then, it explains how the hyperparameters of the models are determined and how the models themselves were trained. This study tested four different regression models and three classification methods.

#### 4.3.1. Regression Models

In our approach, we wanted to evaluate whether we can use regression models for predicting subjective exercising fatigue. We used the extracted AUs as an input feature to predict the target value heart rate.

##### Linear Regression

Linear regression trains a linear weighting of the input features. This means that the weight vector $\vec{w}$ is chosen such that the error of the prediction $\hat{y}=w_{0}+w_{1}x_{1}+\cdots +w_{n}x_{n}$ becomes minimal. Linear regression is a fairly simple model that has no hyper parameters [34].

##### Polynomial Regression

Polynomial regression is also a linear model. However, the dimension of the input vector is initially increased by forming the polynomials of the degree *n* of the input features. For example, the vector ${\left(\begin{array}{cc} a & b \end{array}\right)}^{T}$ becomes the vector ${\left(\begin{array}{cccccc} 1 & a & b & a^{2} & ab & b^{2} \end{array}\right)}^{T}$. As a result, the prediction is no longer linear to the input features but to the polynomials of the input features.

To keep the individual weights low, ridge regression is used, which punishes high weights during learning. This way, polynomial regression has two hyperparameters. The first one is the degree *n* of the polynomials, and the second one is the regulatory strength α, which indicates how the ridge regression penalizes high weights.

##### Support Vector Regression

Furthermore, it was used the support vector regression together with an RBF kernel, which makes it possible to approximate non-linear functions. The support vector regression works similarly to the support vector classification, but the support vectors are not used to divide data points into classes but to put a hyperplane through the points. Like the Support Vector Classification, the Support Vector Regression uses two hyperparameters. The hyperparameter γ influences the width of the kernel, while the hyperparameter *C* is used for regularization.

##### Decision Tree Regression

Decision trees are based on a series of queries, which are arranged in a tree-like structure. Each node that is not a leaf represents a query. Depending on whether an input vector fulfills the criteria of the query or not, the path continues to the right or left of the node. The leaves of the tree represent one or more data points of the training data set. To predict a data point, the path is followed from queries until a leaf is reached. The predicted value is the average of the data points represented by the corresponding leaf [34]. In this case, it was considered the hyperparameter maximum depth of a path was the most relevant for the model selection.

#### 4.3.2. Classification Models

For the classification task, we wanted to classify whether participants are exhausted or not. We used a BORG value of ten as the classification threshold (i.e., not exhausted <5, exhausted ⩾5) We used the extracted AUs as input feature to classify subjective exhaustion.

##### Logistic Regression

Logistic regression is a linear classification model. It trains a hyperplane that describes a boundary between two classes. Depending on the distance of a data point to the hyperplane, it is classified into one of two classes. The distance function is linear to the features of the input vector. Logistic regression uses a hyperparameter that determines the strength of the regularization [34].

##### Support Vector Classification

The Support vector classification learns which data points are most important to set a boundary between two classes. When predicting a new data point, the distance to some important points, the so-called support vectors, and the influence of the support vectors on the border are considered. To obtain a non-linear boundary, a non-linear kernel can be used to calculate the distance. The RBF kernel was used for this work [34].

The Support vector classification uses two different hyperparameters. The hyperparameter γ influences the width of the kernel and thus how far the influence of the individual support vectors extends. Here, low values correspond to a wide weight, while high values limit the scope of the influence. The second hyperparameter, *C*, is used for regularization and influences how strong the impact of the individual support vectors is on the boundary between the classes [34].

##### Decision Tree Classification

Decision trees can be used not only for regression but also for classification. The classification procedure is the same as for regression, with the only difference that the prediction when a leaf is reached is the class of data points represented by the leaf.

#### 4.3.3. Hyperparameter Tuning

Once explained the essential function of the models are under consideration and what the associated hyperparameters are, it is necessary to discuss how these hyperparameters are determined. For this purpose, it is divided the data into a test set and a training set.

#### Grid Search

Grid search is a simple method to optimize hyperparameters. A list of values is given for each of the different hyperparameters. Grid search trains the selected model with all combinations of values and determines which combination provides the best prediction. The split the previously generated training set again, so that a new training set is created, on which it can train the hyperparameters, and another data set, which is used to validate the hyperparameters. The previous procedure has the disadvantage that the evaluation of the hyperparameters strongly depends on the division into training and validation data sets. Since an unfavorable division could lead to the hyperparameter being rated too good or too bad, it is additionally used cross-validation. Thus, we generate several divisions on which we train a model with the hyperparameters and test it. This procedure makes the training much more robust since the statement about the generalization ability of the hyperparameters becomes more precise. However, the computing effort of the training increases many times over, since significantly more models are trained. In this case, it is used three different strategies for the data set sampling.

#### 4.3.4. Data Set Sampling

##### k-Fold and Stratified k-Fold

The data is divided into k approximately equal, pairwise disjoint partial data records. If k-fold is used for the subdivision for cross-validation, one of the partial data sets is selected in turn as a test data set, while the other partial data sets are used for training. In the context of this work, the data must be mixed before the division, since the partial data set otherwise contains all data points of individual recordings, while data points of other recordings are not included. If the model being trained is a classification model, it is used stratified k-Fold. This method divides the data set, as well as, k-fold into k partial data sets, but also ensures that the proportions of the individual classes in the partial data set are the same.

##### Group k-Fold

Group k-Fold also divides the data into k partial data sets, which are about the same size. In addition to the data points, the group k-fold specifies groups to which the data points belong. Group k-fold selects whole groups when dividing them up so that it can be examined how the trained model affects unknown groups. Though, this procedure is unnecessary in the context of this work since the images of individual people can be explicitly selected for the training. This makes it possible to assess whether the trained model can also make good predictions for previously unknown people.

##### Shuffle Split and Stratified Shuffle Split

The third split method considered is the shuffle split. It first mixes the data record and then divides it into training and test data. The difference to the k-fold is that the size of the training and test data set, as well as the number of repetitions, can be freely selected. This method was used in this work to test how good the predictions of the trained model are when the training data set is significantly smaller than usual. As with the k-fold, there is a stratified version of the shuffle split, which ensures the same proportions of the different classes in the individual partial data records.

### 4.4. Action Unit Dimension Reduction

**Figure 8:**
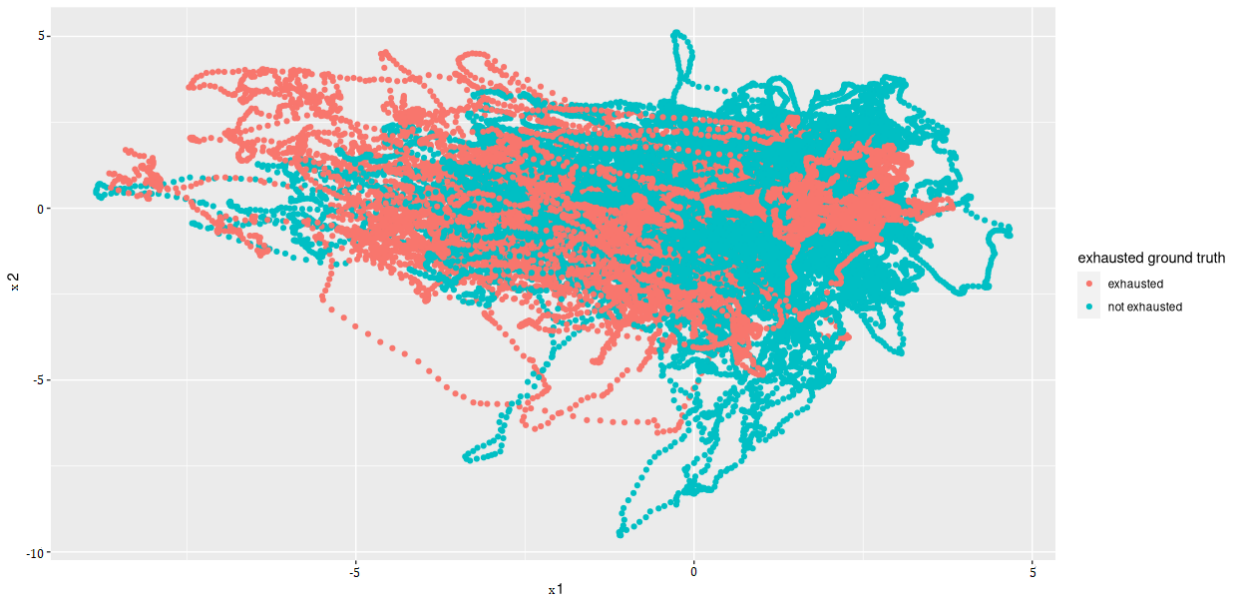
Principal Components 1 and 2 after the dimensionality reduction.

Principal Component Analysis to reduce the computing time and to uncorrelated the feature data to one another is used. Since the features that have the least variance and, therefore, the least information are omitted when reducing the dimensions, a type of smoothing also takes place. Before PCA was applied, the individual features were standardized so that they had an average of zero and a variance of one. The PCA showed that the first 12 of 17 components represent around 93% variance. Thus, we selected a dimension reduction on these 12 components (see Figure 8 for an example plot).

### 4.5. Model Evaluation

The training process is divided into two steps. First, the hyperparameters were determined using a grid search with cross-validation within the search and evaluated on a single test data set. Then, cross-validation is carried out to get a better insight into the generalization properties of the model with the calculated hyperparameters on the entire data. The disadvantage of this approach is that the strict separation of training and test data is sometimes lost. In the k-fold cross-validation, every data point is in one of the k iterations in the test data set, and parts of the test data set were included in the decision when determining the hyperparameters. However, it assumes that this has a negligible impact on the performance of the trained model, as a maximum of two hyperparameters is affected. Some tests confirm this statement. In most cases, the cross-validation performance was even slightly worse than that of the grid search test data set.

## 5. Evaluation

In this chapter, the results of the models under consideration will be discussed in more detail. For this purpose, the different model types are first considered and compared with each other. The influence of the various steps in the pipeline is then examined using a single model.

### 5.1. Metrics

Various metrics were considered for the evaluation. The regression models were optimized based on the coefficient of determination $R^{2}$. In addition, the mean absolute error (MAE) was calculated, since the meaning of this error measure is much more intuitive. In the context of this work, an MAE of, for example, .1 means that the prediction deviates on average by one category on the Borg CR10 scale. The classification models were optimized based on the $F_{1}$ score, which weighs the metrics’ precision and recall and summarizes them in a single value. This metric was chosen because it describes the capabilities of a model better than the accuracy score, especially in the case of unevenly distributed classes [34]. Since accuracy is much more intuitive, it will also be considered below. Finally, we will also report precision and recall because in the application under consideration it is better to make an error of the 2nd type than an error of the 1st type. The system tries to inspire the user, even though the person is not yet exhausted, this could lead to user frustration. However, if the system fails to inspire an exhausted person, the quality of the training is reduced.

### 5.2. Model Comparison

**Figure 9:**
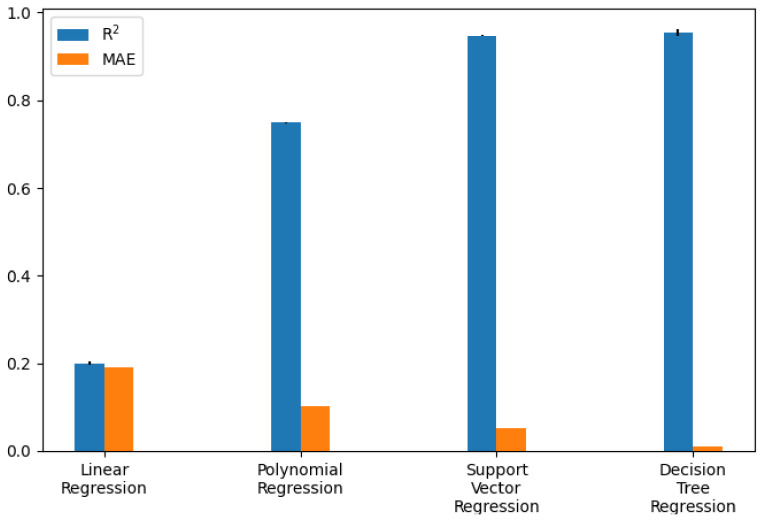
Regression model performance as bar chart (exact values are in Table 5).

**Figure 10:**
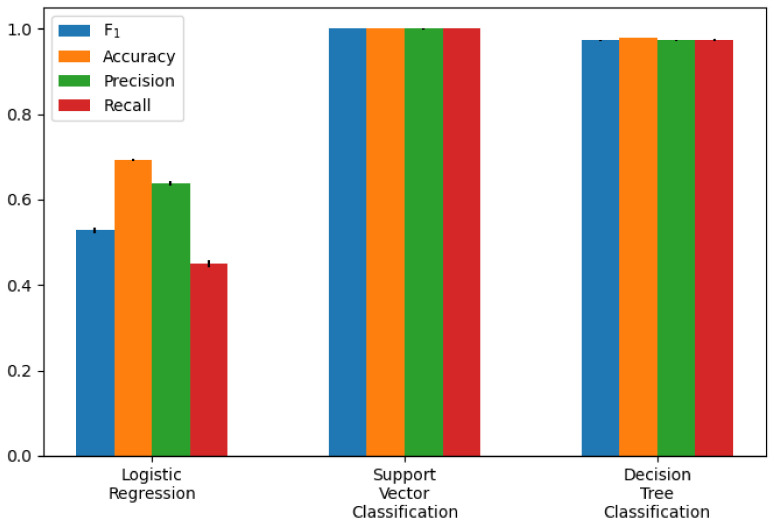
Classification model performance as bar chart (exact values are in Table 6).

**Figure 11:**
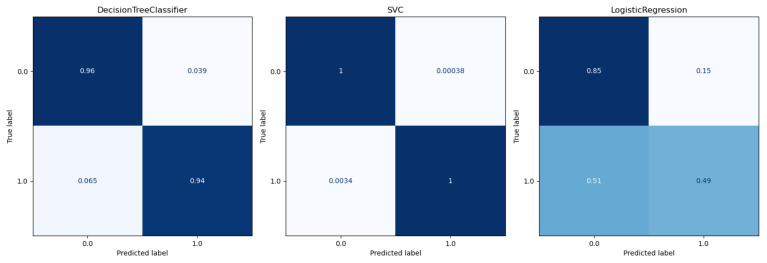
Confussion matrix for classifciation models.

**Figure 12:**
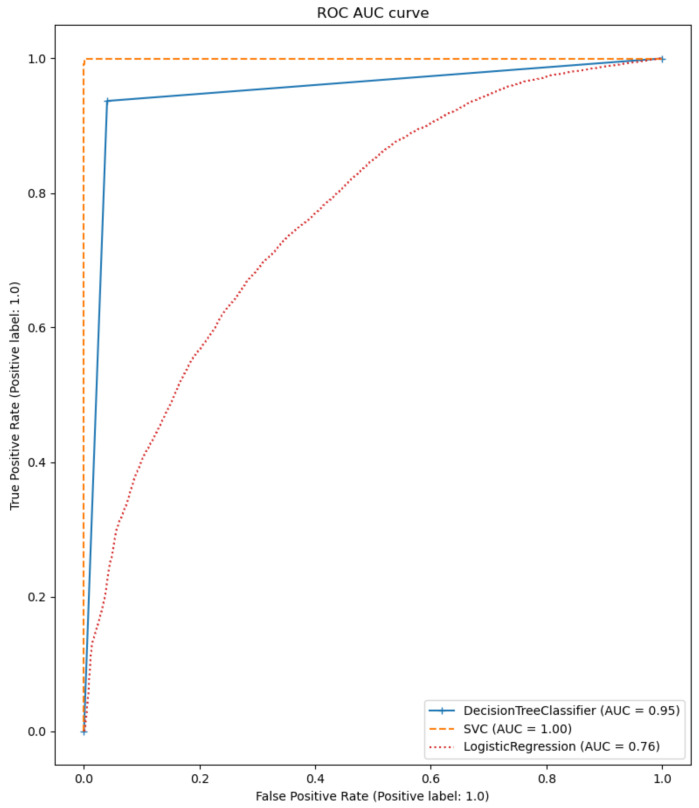
ROC-AUC plot for classification models.

The performance of the different models was compared as the first step. The participants who had a *rating* of at least three and a *smiling* value of zero were selected. An average filter with a convolution kernel of width 49 (this corresponds to approximately 2 s) was used to smooth the AU data. To split the data sets, k-fold or stratified k-fold with k = 5 was used. The results of the calculated metrics are shown in Table 5 and Table 6. The average of the values calculated in the cross-validation was given. A bar chart of the metrics was also used for better comparability. The two bar graphs can be seen in Figure 9 and Figure 10. In addition, one example of the AUC-ROC curve and confusion matrix is depicted in Figure 11 and Figure 12 respectively.

**Figure 13:**
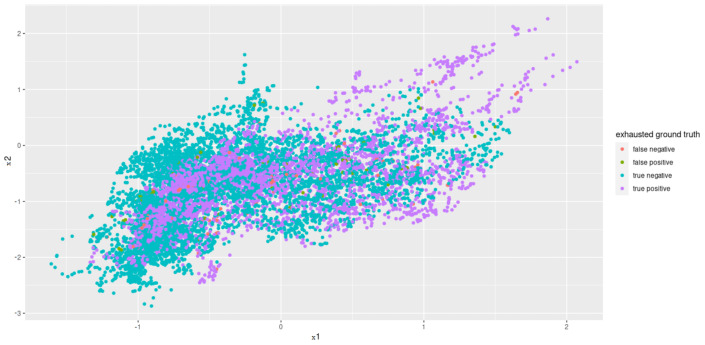
Principal components 1 and 2 for results of best classification model (DTC) after grid search.

The calculated metrics show that the data can only be poorly represented by a linear model. This can be seen in the results of logistic regression, but above all, in those of linear regression. The performance of the non-linear models is significantly better. Both the results of the decision trees and the support vector machines are better than originally expected (see Figure 13 for the best classification model). This suggests that there may be a strong correlation between a sports person’s facial expression and their level of exhaustion. Since the fatigue values of the Borg scale were scaled to a value between zero and one, the MAE of the Decision Tree Regression means that the predicted Borg value has an average deviation of only 0.106.

### 5.3. Influencing Variables

After comparing the basic performance of the models, the individual steps of the pipeline were examined in more detail. For this purpose, the steps of the pipeline in the previous paragraph were changed individually. To be able to consider as many steps of the pipeline as possible, the modified pipeline was only evaluated on a single regression model, since consideration of all models would exceed the scope of this work. The Decision Tree Regression was chosen for a closer look because it delivers good results and can be calculated much faster than the Support Vector Regression.

#### 5.3.1. Participant Selection

It is noticed that the model has difficulties predicting grinning participants, as we assumed earlier. For this purpose, different intervals for the values *rating* and *smiling* were examined in the selection step. The results of this are shown in Table 7.

The calculated metrics show that the decision tree regression makes poorer predictions when both grinning and non-grinning people are considered. However, the model’s performance is still good, so the approach seems to be suitable for prediction. Surprisingly, the performance drops only slightly if you only look at people who grin while doing the exercises. This suggests that the influence of the grin can be offset by other facial features.

#### 5.3.2. AU Smoothing

Section 4.2.2 explains several methods that can be used to smooth the action unit data. This section looks at the impact of these methods on the quality of the prediction. The results for these metrics are listed in Table 8. The results of the calculated metrics show that the type of smoothing has a large impact on the prediction. In this case, the best results are obtained using a global low pass filter. However, as already mentioned in Section 4.2.2, this has the disadvantage that it is not suitable for real-time applications and is therefore not considered further below. It is striking that the quality of the prediction is directly related to the strength of the smoothing. For example, the MAE becomes more than twice as high when the width of the mean filter is halved. The prediction is best with the mean filter that was chosen because it had the strongest smoothing. The need for smoothing becomes particularly clear in the run without smoothing, in which the model created had very little predictive power.

#### 5.3.3. Data Set Splitting

Finally, it was considered how the strategy for dividing the data set affects the quality of the forecast. The metrics calculated for this are shown in Table 9. The metrics of the run with the k-fold show that this strategy is only very poorly suited to dividing the data. This means that the procedure used is not suitable for predicting the degree of exhaustion of a person who is not included in the training data set. The results of the shuffle split show that the trained model can be generalized well for well-known people. Even if the model is only trained on 25% of the data, the statements come very close to the actual degree of exhaustion. If the training data set consists of 50% of the data, the accuracy increases again significantly.

## 6. Discussion

This work aimed to investigate the feasibility of predicting perceived exhaustion in STS training based on facial action units. We collected a novel data set that included video recordings of 60 participants in the aforementioned exercise routine. Each subject provided a subjective exhaustion measurement during the training, which was used as the target variable to predict. As predictor variables, the extracted AUs from the recordings were employed. After cleaning and pre-processing the data set, we fitted different regression and classification models that should predict the perceived exhaustion based on the exercisers’ AUs. The results show that the decision tree model, as well as the support vector model, provide high prediction results on train and validation sets.

Compared to related work in this area [23,29], this work was focused on the problem of estimating and classifying perceived subjective exhaustion using the FACS in a machine learning framework. Systematically, it was compared different machine learning models to assess the accuracy of predicting perceived exhaustion. Thus, not only looked at linear regression models but evaluated support vector and decision tree methods as well. In this case, it was investigated the impact on accuracy based on the preprocessing (e.g., smoothing), participant’s responses to the training (e.g., smiling), and the models using shuffled evaluation sets with previously unseen data for the model were tested.

Therefore, we think that our research fills a gap in the literature and further supports previous work on the possibility to predict perceived exhaustion using the FACS. Having a non-obtrusive exhaustion recognition system could come in handy for the deployment of coaching systems that should help users to keep motivation high during exercise training. Even though the system might not perform well on the first encounters, it could adapt to the user. Therefore, applied research is required, which evaluates pre-trained machine learning models for exhaustion prediction in the real world. Consequently, further limitations and challenges have to be tackled, which we will explain in the following.

### Limitation

The influence of different variables on the models’ predictive power was investigated. This work shows that the approach presented here is limited since it relies on having seen the participant. Despite this, the participants have encountered interaction with an artificial coach. Thus, the model can be retrained after it has seen examples from a specific user.

It is necessary to have into account that during preprocessing the video files, it was noticed that the behavior of the different participants was distinct. While some participants had strained facial expressions at the end of the exercise, others had neutral facial expressions throughout the practice. Furthermore, it happened with some participants that they started to grin during the activities, which is probably due to the unusual situation. Including an automatic smile recognition in our pipeline could be used as an additional feature that could be automatically fed into the model retraining. This could additionally help to make the model robust for individual differences and incorporate them.

Regarding the goal of transferring this approach to a real-time system, at least 25 fps should be processed, whereby constant delays are permitted. Runtime measurements of the individual steps have shown that this goal could not be met. While the calculations of the mean value filter and the prediction are sufficiently fast, the calculation of the AUs takes too much time. With the implementation that accompanies this work, only 4.5 images could be processed in 1 s However, since the article presents and describes OpenFace as a real-time application, there is a possibility that slow processing is due to underperforming hardware or inefficient use of the library.

A major limitation of the approach used in this work is the limited sample size. Conducting this kind of data collection with the subjects is cost-intensive. However, enlarging the sample size could enable researchers to investigate additional models that require even more training data (e.g., neural networks). While we also could generate high predictive outputs in the cross-evaluation step, we could not show that the models work efficiently with unseen participants. Thus, our models seem to over-fit previously seen subject behavior. Though, as mentioned earlier, it was also anticipated that retraining the model is a suitable approach to overcome this issue. Thus, future work includes researching a validation strategy where we leave one participant out, train the model, and retrain it on the left-out sample. This could shed light on whether this approach truly works with samples from a first encounter with the system.

Furthermore, the data collected was from a highly restricted experiment. Results might be very different when collecting data ’in-the-wild’, without giving the participants any restrictions on how they have to move or interact. The same holds for the controlled exercise. In this case, the participants that did an STS exercise were the evaluated population. This could indicate that different results when participants conduct stationary bicycle exercises or rowing movements can be present.

While there are many things open for research, we assume that this approach is an exciting research area to follow, as there are few works that have looked at how to predict perceived exhaustion based on facial features.

## 7. Conclusions

This work presents the development and evaluation of different computational models to estimate the user’s fatigue state (exhausted and not exhausted) for the sit-to-stand exercise. Hence, a study with 60 healthy people was carried out, to record the test and to obtain the training and test data. The models are based on 17 facial actions measured by using the FACS, the volunteers’ reported Borg values as fatigue state reference, and machine learning techniques. Results show that after a rigorous preprocessing procedure of the videos and data, it is possible to train a support vector classification model and a Decision Tree classification model with accuracy and precision higher than 0.97, and a mean absolute error (MAE) lower than 0.003.

The preprocessing which consisted of cleaning the images from noise, covering the faces of not desired people, smoothing the AU face measurements through digital filters, and implementing Principal Component Analysis (PCA) reduces the computing time and splits the data records to set the data sampling. Furthermore, the videos were rated regarding the time that the subject was smiling and how easy is to recognize his/her facial expressions, to use the videos that contain relevant information.

Finally, an analysis process was executed to determine the impact of the filter and the splitting data strategy regarding the model prediction performance metrics, by changing the filter width (Table 8) and testing 3 different sampling strategies (Table 9). Results suggest that when the AU measurements are not smoothed, the classification exhaustion models present a little predictive power, due to the volunteer face movement during the test that does not allow a clear AU estimation. On the other hand, results suggest that the Shuffle Split strategy can be used to train a model able to generalize well the exhaustion state of known people, even if it is only trained with 25.

The main limitation is the limited sample size. However, contrasting to similar works, this work presents the problem of estimating and classifying perceived subjective exhaustion using the FACS in a machine learning framework, by comparing different machine learning models. Besides, it is also studied the impact of the preprocessing methods, on the model prediction performance. Therefore, this research fills a gap in the previous literature and further supports previous work on the possibility to predict perceived exhaustion using the FACS.

## Figures and Tables

**Table 1:** Volunteer descriptive data (M ± SD).

Gender	Age (Years)	Weight (kg)	Height (cm)
f	20.8±1.7	59.3±5.5	164.1±7.7
m	21.9±1.9	65.9±6.4	172.8±8.3

**Table 2:** AUs extracted with OpenFace.

Name Action Unit	Facial Action Coding Name
1	Inner brow raiser
2	Outer brow raiser
4	Brow lowerer
5	Upper lid raiser
6	Cheek raiser
7	Lid tightener
9	Nose wrinkler
10	Upper lip raiser
12	Lip corner puller
14	Dimpler
15	Lip corner depressor
17	Chin raiser
20	Lip stretcher
23	Lip tightener
25	Lips part
26	Jaw drop
45	Blink

**Table 3:** Annotation of *smiling*.

*Smiling* Value	Meaning
0	participant does not smile
1	participant smiles sometimes
2	participant smiles most of the time

**Table 4:** Annotation of *rating*.

*Rating* Value	Meaning
1	data is not usable (e.g., participant looks seldom to the camera)
2	participant does not look exhausted
3	participant has a neutral face, or switches between looking exhausted or not exhausted
4	participant looks slightly exhausted
5	participant looks very exhausted

**Table 5:** Accuracy of evaluated regression models. Optimized hyperparameters are in brackets.

Model	$R^{2}$	MSE	RMSE	MAE
Linear Regression	0.20	0.05	0.22	0.19
Polynomial Regression (*n* = 3, α = 10)	0.74	0.001	0.03	0.1
Support Vector Regression (*C* = 1000, γ = 0.1)	0.94	0.003	0.05	0.05
Decision Tree Regression (depth = 28)	0.95	0.003	0.05	0.01

**Table 6:** Accuracy of evaluated classification models. Optimized hyperparameters are in brackets.

Model	F1	Accuracy	Precision	Recall
LogReg (*C* = 1)	0.53	0.69	0.64	0.45
SVC (*C* = 100, γ = 1)	0.99	0.99	0.99	0.99
DTC (depth = 22)	0.97	0.97	0.97	0.97

**Table 7:** The impact of participant’s behavior on prediction accuracy for Decision Tree Regression.

Selection	$R^{2}$	MSE	MAE
rating ∈ [3, 5], smiling = 0	0.95	0.003	0.01
rating ∈ [3, 5], smiling ∈ [0, 2]	0.92	0.005	0.01
rating ∈ [3, 5], smiling ∈ [1, 2]	0.94	0.003	0.01
rating ∈ [1, 5], smiling ∈ [0, 2]	0.91	0.005	0.01
rating ∈ [1, 3], smiling ∈ [0, 2]	0.92	0.005	0.01

**Table 8:** The impact of data preprocessing on prediction accuracy for Decision Tree Regression.

Smoothing	$R^{2}$	MSE	MAE
moving average filter w = 49	0.95	0.003	0.012
moving average filter w = 25	0.87	0.009	0.027
binomial filter w = 49	0.667	0.023	0.06
golbal low pass filter $F_{max}$ = 0.02	0.976	0.002	0.005
local low pass filter w = 49, $F_{max}$ = 1	0.828	0.012	0.033
no smoothing	0.234	0.055	0.18

**Table 9:** The impact of data set splitting on prediction accuracy for Decision Tree Regression.

Sampling Strategy	$R^{2}$	MSE	MAE
k-Fold k = 5	0.95	0.003	0.012
Group k-Fold k = 5	−0.28	0.08	0.23
Shuffle Split (25%)	0.84	0.011	0.034
Shuffle Split (50%)	0.92	0.005	0.017

## Data Availability

Publicly available datasets were analyzed in this study. This data can be found here: https://github.com/sebschne/sensors-22-06524, accessed on 9 February 2022.
